# Elevated Serum GAD65 and GAD65-GADA Immune Complexes in Stiff Person Syndrome

**DOI:** 10.1038/srep11196

**Published:** 2015-06-16

**Authors:** Gucci Jijuan Gu Urban, Mikaela Friedman, Ping Ren, Carina Törn, Malin Fex, Christiane S. Hampe, Åke Lernmark, Ulf Landegren, Masood Kamali-Moghaddam

**Affiliations:** 1 Dept. of Immunology, Genetics and Pathology, Science for Life Laboratory, Uppsala University, Uppsala, Sweden; 2 Dept. of Clinical Sciences, Skåne University Hospital SUS, Lund University, Malmö, Sweden; 3 Dept. of Medicine, University of Washington, USA; 4 Dept. of Genetics, Stanford University, CA 94305, USA

## Abstract

Glutamic acid decarboxylase 65 (GAD65) and autoantibodies specific for GAD65 (GADA) are associated with autoimmune diseases including Stiff Person Syndrome (SPS) and Type 1 diabetes (T1D). GADA is recognized as a biomarker of value for clinical diagnosis and prognostication in these diseases. Nonetheless, it remains medically interesting to develop sensitive and specific assays to detect GAD65 preceding GADA emergence, and to monitor GADA-GAD65 immune complexes in blood samples. In the present study, we developed a highly sensitive proximity ligation assay to measure serum GAD65. This novel assay allowed detection of as little as 0.65 pg/ml GAD65. We were also able to detect immune complexes involving GAD65 and GADA. Both free GAD65 and GAD65-GADA levels were significantly higher in serum samples from SPS patients compared to healthy controls. The proximity ligation assays applied for detection of GAD65 and its immune complexes may thus enable improved diagnosis and better understanding of SPS.

Autoantibodies against glutamic acid decarboxylase 65 (GAD65) are found in a majority of patients with Stiff Person Syndrome (SPS)^1^,^2^. The appearance of autoantibodies specific for GAD65 (GADA) precedes and predicts the development of SPS and other GADA-associated autoimmune diseases such as type 1 diabetes (T1D)^3^. Accordingly, GADA serves as a diagnostic biomarker for SPS (positive in 70–80% of patients)^4^. Because of the cytosolic location of GAD65, GADA production may only be triggered after release of GAD65. Major GAD65-expressing cell types are neurons and pancreatic beta cells^5^. It remains to be determined whether GAD65 is released from both of these cell types or from only one of them, and whether the source of the released GAD65 correlates with the different autoimmune condition.

Detection of GAD65 and GAD65-GADA complexes in blood may enable earlier diagnosis, as well as monitoring of the ongoing destruction of targeted cells, provided sufficiently sensitive assays can be developed. In recent years, several relatively sensitive methods have been developed in attempts to detect endogenous GAD65 in blood. The most sensitive immunoassay allowed detection of GAD65 in human plasma spiked with as little as 31 pg/ml recombinant human GAD65 (rhGAD65)^6^, and a time-resolved fluorescence immunoassay detected GAD65 with a limit of detection (LOD) of 100 pg/ml^7^. Neither of these assays was able to detect endogenous GAD65 in sera of SPS or T1D. The insufficient detection sensitivity of the current assays could also be impeded by the short lifetime of GAD65 (1.25–2.37 h in human blood at 37 °C)^8^. Another potential obstacle to accurate and sensitive measurement of GAD65 in human serum is the presence of GADA binding GAD65, which may interfere with accessibility and detectability of GAD65 in blood samples.

Here we have developed proximity ligation assays (PLA) for sensitive and selective measurement of both GAD65 (Fig. 1A) and of GAD65-GADA complexes (Fig. 2A) in human serum. The PLA technique has been used for high-performance protein detection both in single-9,10,11 and multiplex^12^,^13^,^14^ assays, and for detection of more complex targets such as microvesicles^15^. In solid-phase PLA, target proteins are first captured by an immobilized antibody, next, pairs of antibodies are added, that have been conjugated with distinct oligonucleotides, referred to as PLA probes. Upon binding of a pair of PLA probes to a captured protein molecule or molecular complex, the DNA strands are being enzymatically ligated, guided by a connector DNA strand. This ligation reaction generates a DNA template that can be amplified and quantified by quantitative PCR as a measure of the amount of proteins.

## Results

### Detection of GAD65 using solid-phase PLA

**Figure 1:**
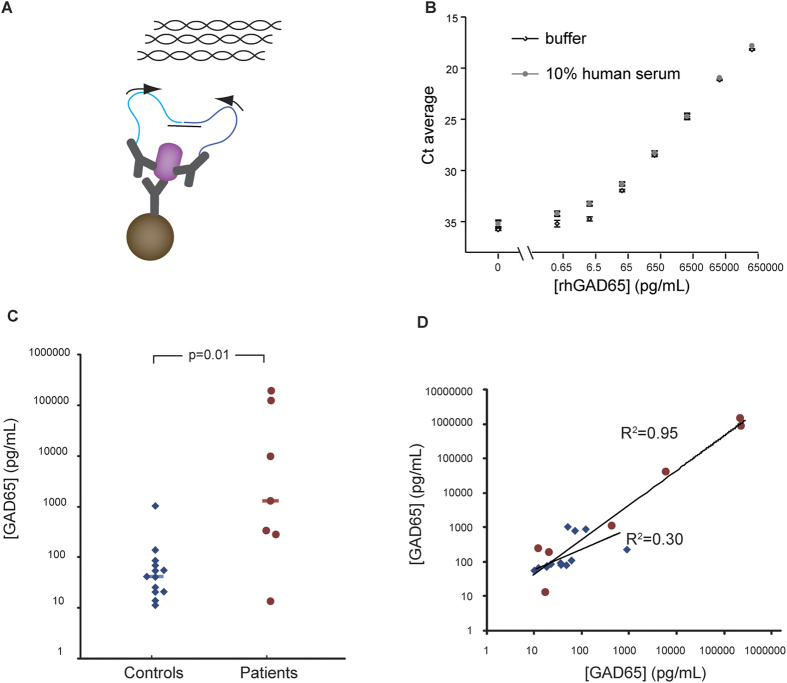
Detection of GAD65 by solid-phase PLA. **A**) Goat polyclonal anti-human GAD65 antibodies were covalently coupled to magnetic beads for capturing GAD65 proteins from biological samples. Two other portions from the same anti-GAD65 polyclonal antibody preparation with distinct attached oligonucleotides, referred to as PLA probes, bound to GAD65 captured on the magnetic beads. Upon hybridization of a connector oligonucleotide the two antibody-conjugated DNA oligonucleotides were enzymatically joined to serve as an amplification template, and quantified by qPCR. **B**) Solid-phase PLA was used to detect recombinant human GAD65 (rhGAD65) in a dilution series (65 ng/ml – 0.65 pg/ml) in 10% control human serum and assay buffer. The limit of detection was approximately 0.65 pg/ml. Averages from triplicate measurements are shown with error bars that indicate the standard deviation. **C**) Levels of GAD65 were compared in serum samples from SPS patients and controls. Significantly increased GAD65 levels (p = 0.01) were observed for the SPS group (n = 7) compared to the controls (n = 13). The horizontal bars indicate the median for the patient and control groups. The assays were performed in three replicates. **D**) Scatter plot showing the correlation between two independent measurements of GAD65 in serum samples from SPS patients (R^2^ = 0.95) and controls (R^2^ = 0.30), red circles represent SPS patients and blue diamonds are controls.

We established an assay for detection of endogenous GAD65 in human serum using solid-phase PLA (Fig. 1A). To compare the assay performance in buffer and the complex biological matrix of human serum, we detected rhGAD65 in PLA buffer or 10% control human serum prepared in PLA buffer that was spiked with different concentrations of rhGAD65 (from 65 ng/ml to 0.65 pg/ml and a blank) (Fig. 1B). The assay performance in 10% control human serum was comparable to that in PLA buffer. Both assays provided a LOD of approximately 0.65 pg/ml with a broad dynamic range of up to six orders of magnitude. Because the assay required only 5 μl sample per reaction, the LOD corresponded to 3 fg GAD65 (approximately 30,000 molecules) per investigated sample. The minimal detectable amount of GAD65 was approximately 460-fold lower than that of the most sensitive GAD65 detection assays previously reported (30 pg/ml, 50 μl sample/reaction)^8^.

Next, we applied the assay to measure endogenous GAD65 proteins in serum samples from seven patients diagnosed with SPS, and thirteen healthy controls (Supplementary table 1). Significantly elevated levels of GAD65 were observed in the samples from SPS patients (median, 1.129 ng/ml; range 13.2 pg/ml-1.513 ng/ml) compared to those from the control group (median, 43 pg/ml; range 11.3 pg/ml-1.036 ng/ml, p = 0.01) (Fig. 1C. Supplementary table 1). The linear correlation coefficient (R^2^) between two independent measurements of GAD65 in the SPS samples was 0.95 (Fig. 1D), reflecting excellent reproducibility of the assay in patient samples. The R^2^ between two measurements of GAD65 in the control group was 0.30 (Fig. 1D). The lower correlation is possibly a consequence of the undetectable or trace amounts of GAD65 in the control samples. The average intra-assay variability in two independent analyses was 30% (19% for SPS samples; 42% for controls) and 13% (11% for SPS samples; 15% for controls), respectively.

### Detection of complexes of GAD65 and GADA in clinical samples using solid-phase PLA

**Figure 2:**
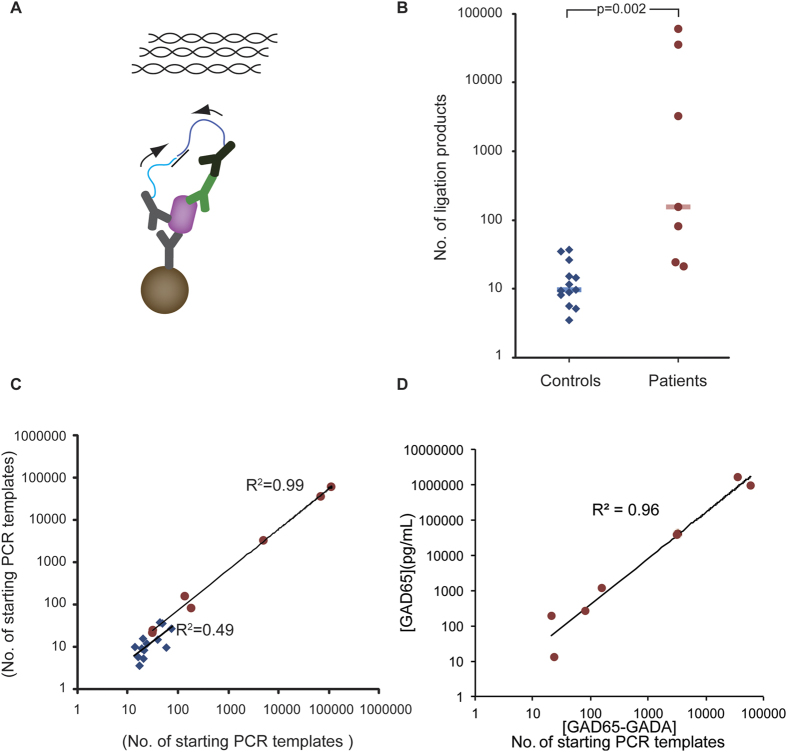
Detection of GAD65-GADA immune complexes using solid-phase PLA. **A**) GAD65-GADA complexes was detected similarly to the measurement of GAD65 using solid-phase PLA, but replacing one of the PLA probes specific for GAD65 with one directed against human IgG 1. **B**) Solid-phase PLA was used to measure GAD65-GADA immune complexes in blood serum samples from SPS patients (n = 7) and control samples (n = 13). Significantly increased levels of GAD65-GADA complexes were recorded in the SPS group (median number of PCR starting amplicons 155 (red bar)) in comparison with the control group (median No. of PCR starting amplicons 10 (blue bar)) (p = 0.002). **C**) Scatter plot showed the correlation between two independent measurements of GAD65-GADA complexes as calculated numbers of starting PCR amplicons in the SPS group (R^2^ = 0.99, red circles) and the control group (R^2^ = 0.49, blue diamonds). **D**) Scatter plot presenting the correlation between measurements of GAD65-GADA complexes and GAD65 in samples from SPS patients (R^2^ = 0.96).

The requirement in solid-phase PLA for recognition of target proteins by three antibodies serves not only to enhance assay specificity but it also provides an opportunity to study protein-protein interactions. We investigated levels of GAD65-GADA complexes in serum samples from the above patients and controls via solid-phase PLA. In order to measure endogenous GAD65-GADA complexes in human serum, we used the same polyclonal GAD65 antibodies as capture antibodies and for one of the PLA probes, while a donkey antibody against human IgG1 was used for the other PLA probe to detect bound GADA (Fig. 2A). In the absence of a standard curve for quantification of GAD65-GADA complexes in human serum, we converted Ct values from measurements of GAD65-GADA complexes to numbers of starting PCR amplicons (the formula used to calculate the numbers of amplicons is described in Methods and Materials). Average levels of GAD65-GADA complexes were significantly elevated (p = 0.002) in SPS patients (median of PCR starting templates, 155; range 21–60180) compared to the control group (median of PCR starting templates, 10; range 4–37) (Fig. 2B, Supplementary table 1). The linear correlation coefficient (R^2^) between two independent measurements of GAD65-GADA complexes in the SPS samples was 0.99, demonstrating excellent reliability of the measured values, whereas the R^2^ value for the two measurements of the control samples was 0.49 (Fig. 2C). The average intra-assay variability was 29% (25% for SPS samples; 33% for controls) and 17% (14% for SPS samples; 20% for controls), respectively, in two independent measurements of GAD65-GADA immune complexes. Measurements of GAD65 correlated strongly with those of GAD65-GADA complexes among the patients (Fig. 2D; R^2^ = 0.96).

## Discussion

To the best of our knowledge, this is the first time that levels of endogenous GAD65 and GAD65-GADA complexes have been recorded and proven to be significantly elevated in sera from SPS patients compared to healthy controls. This provides a significant improved detection of GAD65 and GAD65-GADA complexes compared to existing methods, and it will enable studies to gain further insights into this autoimmune disease. In the small-investigated cohort of patients and controls, the GAD65-GADA levels varied over a broad range. Two out of seven patient samples had very low levels of GAD65-GADA complexes, consistent with earlier observations that 15% of SPS patients have only traces of GADA levels^16^. In the previous study, the correlation between levels of GADA as measured by the standard radiobinding assay (RBA)^17^ and levels of GAD65-GADA complexes was limited. The reason for this could be that detection of GAD65-GADA complex levels may be dependent on the equilibrium between GADA and available and detectable GAD65 in the samples rather than by the GADA levels alone^8^.

In conclusion, we have developed highly sensitive proximity ligation assays for detecting GAD65 and GAD65-GADA complexes in human serum. The group of SPS patients showed significantly elevated levels of GAD65 and GAD65-GADA complexes compared to a group of controls. Circulating GAD65 and GAD65-GADA complexes are thus both promising biomarkers for SPS. The assays developed in the present study may also prove valuable for prediction and early diagnosis of more prevalent autoimmune diseases such as T1D. Specifically, these assays may allow detection and quantification of GAD65 and GAD65-GADA complexes in prediabetic subjects^18^ or in patients recently diagnosed with T1D, in order to estimate the loss of beta cells once insulin therapy has been initiated^19^. The assays could also prove suitable to monitor GAD65 release associated with beta cell loss in patients transplanted with human pancreatic islets. These patients are at risk of losing the graft as reappearance of GADA has been documented in islet transplanted patients^20^, GAD65 and GAD65-GADA assays may be used to monitor loss of grafted islets prior to the reappearance of GADA. Both assays may also prove suitable to monitor therapeutic interventions, and for screening of drug libraries.

It remains unclear what triggers the destruction of pancreatic beta cells and the subsequent release of beta cell antigen in T1D, but viruses killing the beta cells is considered as one possible mechanism^21^. The destruction of the beta cells likely initiates an autoimmune reaction, an immune response that is reflected by the appearance of autoantibodies against beta cell antigens including insulin and GAD65. The presence of circulating GAD65 in blood may serve as an early marker for beta cell destruction. As GADA develop, the appearance of GAD65-GADA complexes may reflect progressive loss of beta cells. These hypotheses can now be tested with the present assays using samples collected from birth in children at increased genetic risk for T1D such as in the ongoing TEDDY study^22^.

## Material and Methods

### Clinical samples

Serum samples were obtained from seven adults diagnosed with SPS (Median age, 50; Range, 34–71 years old; Gender, male: 3/female: 4). As a control group 13 healthy blood donors were used. All experiments were performed in accordance with relevant guidelines and regulations, and local institutional ethics committee approval and subjects’ consent were obtained prior to collection of all serum samples (University of Washington, Seattle, USA; Lund University, Sweden). The SPS samples were measured for GAD65 autoantibodies (GADA) using a radiobinding assay as previously described^23^ (Supplementary table 1).

### Proteins and oligonucleotides

Polyclonal goat anti-human GAD2/GAD65 antibodies (directed against amino acid 1–150) (AF2247, R&D Systems) and donkey anti-human IgG antibodies (709-005-149, Jackson ImmunoResearch, West Grove, PA, USA) were used as PLA probes after conjugation to oligonucleotides. Recombinant human GAD65 (rhGAD65) protein was purchased from Diamyd (T-cell GAD65, 10-65702-15-01, Diamyd Medical, Sweden) and used to produce a standard curve for rhGAD65 and to validate the PLA probes. DNA oligonucleotides for preparation of PLA probes were modified with a thiol group at the 5′ or 3′ end (5′ SH-CGCATCGCCCTTGGACTACGACTGACGAACCGCTTTGCCTGACTGATCGCTAAATCGTG-3′ OH) and (5′ P-TCGTGTCTAAAGTCCG TTACCTTGATTCCCCTAACCCTCTTGAAAAATTCGGCATCGGTGA-3′ SH), the latter oligonucleotide having a 5′-phosphate group (Eurogentech). Forward (5′-CATCGCCCTTGGACTACGA-3′) and reverse (5′-GGGAATCAAGGTAACGGACTTTAG-3′) primers for PCR, and a connector oligonucleotide (5′-TACTTAGACACGACACGATTTAGTTT-3′) to guide ligation of the two DNA oligonucleotides were purchased from Biomers. TaqMan probes (5′ FAM-TGACGAACCGCTTTGCCTGA-3′ MGB) were purchased from Applied Biosystems (Grand Island, NY, USA).

### Preparation of PLA probes

DNA oligonucleotides were conjugated to antibodies essentially as described previously^24^. Goat anti-human GAD65 antibodies (20 μg) was dissolved in PBS at a final concentration of 2 mg/ml and incubated for 2 h at room temperature (RT) with a 25-fold molar excess of sulfosuccinimidyl-4-(N-maleimidomethyl) cyclohexane-1 carboxylate (sulfo-SMCC; Pierce, Rockford, IL, USA), freshly dissolved in DMSO. In parallel, 400 pmol of each oligonucleotide modified with a thiol group at either the 3′ or 5′ end was reduced in 20 mM DTT for 1 h at 37 °C. Excessive sulfo-SMCC and DTT were removed from reactions with Zeba spin desalting columns (7K MWCO, Pierce) that had previously been equilibrated with 5 mM EDTA in 1xPBS according to the manufacturer´s protocol. After purification, antibodies and DNA oligonucleotides were mixed at a molar ratio of 1:3 and dialyzed overnight in Slide-A-Lyzer MINI dialysis units (7K MWCO, Pierce) against PBS.

For detection of GAD65-GADA complexes, the PLA probe directed against human IgG was prepared by biotinylating anti human IgG antibodies and coupling these to streptavidin previously conjugated with the appropriate oligonucleotide (Solulink, San Diego, CA, USA). Biotinylation was performed using the ChromaLink™ Biotin Labeling Kit according to the manufacturer’s instructions (B-9007-105K; SoluLink). Successful biotinylation was ascertained by specific absorbance measurement (354 nm) of the traceable biotin-linker. Streptavidin-oligonucleotide conjugates were prepared as previously described^25^. The anti-human IgG PLA probe was prepared by incubating the biotinylated anti-human IgG antibody at a 1:1 molar ratio with 100 nM of streptavidin-oligonucleotide for 1 h at RT.

Prior to use, the anti-human GAD65 PLA probes (GAD65 detection) and the anti-human GAD65 and anti-human IgG PLA probes (GAD65-GADA detection) were mixed at a final concentration of 500 pM of each probe in PLA buffer (0.1% purified BSA (New England Biolabs, pswich, MA, USA), 1 mM D-biotin (Invitrogen, Carlsbad, CA, USA), 0.1 μg/μl salmon sperm DNA (Invitrogen), 4 mM EDTA, 0.05% Tween 20 (Sigma-Aldrich, St. Louis, MO, USA, PBS).

### Coupling of antibodies to microparticles

The antigen-capturing beads were prepared by covalently coupling the goat polyclonal anti-human GAD65 antibodies to magnetic microparticles (Dynabeads M-270 Epoxy; Invitrogen) according to the manufacturer´s protocol, and the beads were kept at a final concentration of 10 mg beads/ml with 5 μg antibodies per mg of beads. The beads can be stored at 4 °C, without loss of activity for up to 5 months.

### Detection of GAD65 and GAD65-GADA by solid-phase PLA

Solid-phase PLA was performed as described previously^25^. For each reaction, 50 μg of GAD65 antigen-capturing beads were washed twice in PBS-0.05% Tween 20 (PBST) and reconstituted in 5 μl of PLA buffer prior to mixing the beads with the sample. RhGAD65 protein was incubated at 65 °C for 2 min and a dilution series in PLA buffer or 10% control human serum (the Blood Center, Uppsala Hospital, Uppsala) was prepared at concentrations ranging from 1 nM to 0.01 pM (65 ng/ml to 0.65 pg/ml). A negative control was included to determine background noise. Patient and control samples were diluted ten-fold in PLA buffer and 45 μl of each diluted sample were mixed with the antigen-capturing beads in 96-well microtiter plates and incubated at 4 °C overnight under rotation. Thereafter, the beads were washed twice with PBST on a 96-well plate magnet (Perkin-Elmer, Waltham, MA, USA). 50 μl of 500 pM PLA probe mix was added to each well and incubated for 1.5 h at 37 °C under rotation. For detection of endogenous GAD65 in clinical samples, PLA probes specific for GAD65 were used. For detection of GAD65-GADA complexes in clinical samples one PLA probe specific for GAD65 and one PLA probe specific for human IgG were employed. Subsequently, the particles were again washed twice with PBST on 96-well microtiter plates. Thereafter, 50 μl of ligation/PCR mix (1xPCR buffer (Invitrogen), 2.5 mM MgCl2 (Invitrogen), 100 nM of each primer, 200 nM TaqMan probe, 0.08 mM ATP, 50 nM connector oligonucleotide, 0.2 mM dNTPs (in which dTTP was substituted by dUTP, Fermentas), 1.5 units Platinum *Taq* Polymerase (Invitrogen), 0.5 units T4 DNA ligase (Fermentas), 0.1 units uracil-N-glycocylase (UNG) (Fermentas)) was added to each well, followed by amplification and quantification of ligation products by quantitative PCR (qPCR). The thermocycling program included an initial incubation for 10 min at 95 °C, followed by 40 cycles of 15 sec at 95 °C and 1 min at 60 °C. Quantitative PCR were performed with an Mx-3000 instrument (Stratagene, La Jolla, CA, USA). The results were presented as threshold cycle (Ct) values, reflecting the amount of PLA ligation products. For clinical samples the Ct values for endogenous GAD65 were converted into weights per volume (pg/ml) by a standard curve of rhGAD65 that was analyzed in the same experiments. For measurement of immune complexes the results were presented as numbers of starting PCR amplicons (as explained below). Each reaction was performed in three replicates in each experiment.

### Data analysis

The qPCR data was analyzed with the MxPro software (Stratagene) and the Ct values were exported and further analyzed with Microsoft Excel software 2010. Limit of detection (LOD) for GAD65 detection was determined as the concentration of GAD65 corresponding to Ct_LOD_ = Ct_N_ – 2xSD where Ct_N_ is average Ct value of background noise and SD is standard deviation of Ct_N_. All data points below LOD were set to LOD at calculation of significant difference for detection of endogenous GAD65 in clinical samples. Ct values of GAD65-GADA complexes were converted to numbers of starting PCR amplicons using the equation N_a_ = 2^(38-Cta)^ where N_a_ is the average number of starting PCR templates for triplicate measurements of sample A using PLA, and Ct_a_ is the average Ct of the sample A. This formula assumes that 38 is the number of PCR cycles required to bring a single PCR template to the threshold for fluorescence detection. The statistical significance between the patient group and the control group regarding levels of GAD65 protein or GAD65-GADA immune complex was calculated using the nonparametric Mann-Whitney U test in R 3.1.2.

## Additional Information

**How to cite this article**: Gu Urban, G. J. *et al.* Elevated Serum GAD65 and GAD65-GADA Immune Complexes in Stiff Person Syndrome. *Sci. Rep.* **5**, 11196; doi: 10.1038/srep11196 (2015).

## Supplementary Material

Supplementary Information
