# Supplementary material for: Elevated Serum GAD65 and GAD65-GADA Immune Complexes in Stiff Person Syndrome
Source: Sci Rep. 2015 Jun 16;5:11196. doi: 10.1038/srep11196 (PMC4468815; doi:10.1038/srep11196)
Supplement: Supplementary Information [file srep11196-s1.doc]

**Supplementary Information for**

## Elevated Serum GAD65 and GAD65-GADA Immune Complexes in Stiff Person Syndrome

**Gucci Jijuan Gu^1,4*^, Mikaela Friedman^1*a^, Ping Ren^1^, Carina Törn^2^, Malin Fex^2^, Christiane S. Hampe^3^, Åke Lernmark^2^, Ulf Landegren^1^ and Masood Kamali-Moghaddam^1¤^**

1) Dept. of Immunology, Genetics and Pathology, Science for Life Laboratory, Uppsala University, Uppsala, Sweden; 2) Dept. of Clinical Sciences, Skåne University Hospital SUS, Lund University, Malmö, Sweden; 3) Dept. of Medicine, University of Washington, USA; 4) Dept. of Genetics, Stanford University, CA 94305, USA

Present address:

^a^Science for Life Laboratory Stockholm (SciLifeLab Stockholm), Sweden

^*^ These authors contributed equally to this work

¤ To whom correspondence should be addressed Dept. of Immunology, Genetics and Pathology, Science for Life Laboratory, Uppsala University, SE-751 22 Uppsala, Sweden. Phone: +46 18 471 4454, Fax: +46 18 471 4808;

E-mail: [masood.kamali@igp.uu.se](mailto:masood.kamali@igp.uu.se)

**Supplementary Table 1.** Characteristics of clinical samples

| Samples | Age | Gender | TID | GAD65  (pg/ml) | GAD65-GADA (ligation products) | GADA**^§^**  (U/ml) |
| --- | --- | --- | --- | --- | --- | --- |
| SPS patients |  |  |  |  |  |  |
| Patient 1 | 46 | Male | Yes | 42015.6 | 3248 | 4000 |
| Patient 2 | 88 | Female | Yes | 1128.7 | 155 | 93821 |
| Patient 3 | 41 | Female | Yes | 1512952.3 | 35507 | 3000 |
| Patient 4 | 54 | Female | Yes | 247.9 | 81 | 11279 |
| Patient 5 | 34 | Male | Yes | 13.2 | 24 | 4348 |
| Patient 6 | - | Female | No | 900726.0 | 60180 | 1567000 |
| Patient 7 | 71 | Male | No | 191.9 | 21 | 17852 |
| Controls |  |  |  |  |  |  |
| Sample 1 | 63 | Male | No | 1035.9 | 26 | - |
| Sample 2 | 63 | Female | No | 20.7 | 5 | - |
| -Sample 3 | 34 | Female | No | 54.2 | 37 | - |
| Sample 4 | 32 | Male | No | 139.3 | 12 | - |
| Sample 5 | 49 | Male | No | 69.4 | 15 | - |
| Sample 6 | 63 | Male | No | 25.6 | 35 | - |
| Sample 7 | 63 | Female | No | 40.8 | 6 | - |
| Sample 8 | 46 | Male | No | 11.3 | 34 | - |
| Sample 9 | 50 | Female | No | 86.2 | 14 | - |
| Sample 10 | 51 | Female | No | 21.0 | 8 | - |
| Sample 11 | 57 | Male | No | 13.9 | 9 | - |
| Sample 12 | 52 | Male | No | 41.8 | 10 | - |
| Sample 13 | 62 | Female | No | 55.5 | 9 | - |

**^§^**The GADA levels in SPS patients were measured by radiobinding assay.
